# Understanding and Attitudes toward Cancer Clinical Trials among Patients with a Cancer Diagnosis: National Study through Cancer Trials Ireland

**DOI:** 10.3390/cancers12071921

**Published:** 2020-07-16

**Authors:** Cathriona Kearns, Ronan Feighery, John Mc Caffrey, Michaela Higgins, Martina Smith, Verena Murphy, Seamus O’Reilly, Anne M. Horgan, Janice Walshe, Ray McDermott, Patrick G. Morris, Maccon Keane, Michael Martin, Conleth Murphy, Karen Duffy, Alina Mihai, John Armstrong, Dearbhaile M. O’Donnell, William M. Gallagher, Ciara M. Kelly, Catherine M. Kelly

**Affiliations:** 1 UCD Conway Institute Dublin, D04V1W8 Dublin, Ireland; William.Gallagher@ucd.ie; 2 UCD School of Medicine, Mater Misericordiae University Hospital Dublin, D07AX57 Dublin, Ireland; profjohnmccaffrey@gmail.com (J.M.C.); mhiggins@mater.ie (M.H.); msmith@mater.ie (M.S.); ciarakelly55@yahoo.com (C.M.K.); 3 Cancer Trials Ireland, Innovation House, Old Finglas Road, Glasnevin, D11KXN4 Dublin, Ireland; ronan.feighery@gmail.com (R.F.); verena.murphy@cancertrials.ie (V.M.); Seamus.OReilly@hse.ie (S.O.); AnneM.Horgan@hse.ie (A.M.H.); Janice.Walshe@tuh.ie (J.W.); RAY.MCDERMOTT@amnch.ie (R.M.); patrickmorris@beaumont.ie (P.G.M.); maccon.keane@mailn.hse.ie (M.K.); michaelmartin@sligo.ie (M.M.); conlethmurphy@gmail.com (C.M.); KarenA.Duffy@hse.ie (K.D.); alina.mihaelamihai@beaconhospital.ie (A.M.); john.armstrong@slh.ie (J.A.); odonnellsec@stjames.ie (D.M.O.); 4 Cork University Hospital, T12DFK4 Cork, Ireland; 5 University Hospital Waterford, X91ER8E Waterford, Ireland; 6 St. Vincent University Hospital, D04YN63 Dublin, Ireland; 7 Adelaide & Meath Hospital Incorporating the National Children’s Hospital (AMNCH), D24KNE0 Dublin, Ireland; 8 Beaumont Hospital, D09A0KH Dublin, Ireland; 9 Galway University Hospital, SW4794 Galway, Ireland; 10 Sligo General Hospital, F91H684 Sligo, Ireland; 11 Bon Secours Hospital, T12DV56 Cork, Ireland; 12 Letterkenny General Hospital, F92FC82 Donegal, Ireland; 13 Beacon Hospital, D18AK68 Dublin, Ireland; 14 St. Luke’s Radiation Oncology Network, St Luke’s Hospital, Rathgar, D06HH36 Dublin, Ireland; 15 St. James’s Hospital, D08W9RT Dublin, Ireland

**Keywords:** cancer, clinical trials, patient perception, understanding

## Abstract

Cancer clinical trials (CCTs) are critical to translation and development of better therapies to improve outcomes. CCTs require adequate patient involvement but accrual rates are low globally. Several known barriers impede participation and knowing how subpopulations differ in understanding of CCTs can foster targeted approaches to aid accrual and advance cancer treatments. We conducted the first nationwide survey of 1089 patients attending 14 Irish cancer centres, assessing understanding of fundamental concepts in CCT methodology and factors that influence participation, to help tailor patient support for accrual to CCTs. Two-thirds (66%) of patients reported never having been offered a CCT and only 5% of those not offered asked to participate. Misunderstanding of clinical equipoise was prevalent. There were differences in understanding of randomisation of treatment by age (*p* < 0.0001), ethnicity (*p* = 0.035) and marital status (*p* = 0.013), and 58% of patients and 61% previous CCT participants thought that their doctor would ensure better treatment in CCTs. Females were slightly more risk averse. Males indicated a greater willingness to participate in novel drug trials (*p* = 0.001, *p* = 0.003). The study identified disparities in several demographics; older, widowed, living in provincial small towns and fewer years-educated patients had generally poorer understanding of CCTs, highlighting requirements for targeted support in these groups.

## 1. Introduction

Cancer Trials Ireland is the largest collaborative cancer research infrastructure in Ireland. Through its network of cancer centres, we conducted the first nationwide study exploring attitudes and understanding of cancer clinical trials (CCTs) among adult oncology patients in Ireland. We also sought to determine factors and supports patients considered important when making decisions about participation. Clinical trials are critical to the translation and development of better oncology therapies. Accrual rates to oncology trials are low globally, with an estimated 1 in 10 clinical trials stopped as a result [1]. In Ireland, a key performance indicator of the National Cancer Strategy 2017–2026 is to increase the percentage of patients on cancer therapeutic clinical trials from 3% to 6% by 2020 [2]. Professional organisations and patient advocacy groups encourage patients with cancer to consider CCTs as a routine component of oncology care [3,4,5].

To achieve an increase in CCT accrual, it is recognised that practical barriers at the site/organisation, physician/provider and patient/community levels must be addressed [6,7,8]. Physicians and providers often have insufficient protocols or those they have exclude significant populations of patients such as the elderly or those with co morbid illnesses. Patients express a willingness to participate in CCTs, yet only small percentages enrol [9]. Cancer trials cannot be conducted unless patients are willing and able to participate. Sociodemographic disparities have shown lower rates of participation in older populations, prompting an announcement by the American National Institute of Health in 2018 for researchers to include participants across the lifespan [10,11,12,13,14].

The Irish Platform for Patients’ Organisations, Services and Industry (IPPOSI) study to ascertain Irish public awareness and understanding of clinical research highlighted poor understanding of the terms “clinical research” and “clinical trial” and that the public while supportive of CCTs were uncomfortable with participating in them [15]. A follow-up study showed greater willingness to participate in non-therapeutic cancer clinical research but less so for therapeutic clinical trials.

We previously conducted a study that identified lack of a CCT option as the main reason for failure to recruit in Ireland [16]. Collectively, these studies showed the need for this current study to identify how patients understood CCTs and what barriers they perceive to participation. Identification of these barriers and efforts to remove them represent critical objectives for cancer investigators, patients, and stakeholders across the research spectrum. Accrual to clinical trials influences the timely availability of new therapies and impacts on patient outcomes. It is important to recognize factors that can influence accrual to clinical trials to offer the opportunity to modify such factors where possible and tailor information to increase accrual in line with Ireland’s National Cancer Strategy.

## 2. Method

This is a descriptive study of patients attending 14 Irish cancer centres from April to November 2016, aged ≥18 years with a clinical diagnosis of malignancy, able to complete a standardised questionnaire independently or with the help of a friend, relative, or oncology staff member. Questionnaire completion implied informed consent. The study received full ethical approval at all participating sites.

The questionnaire was divided into the following sections: (A) demographic details; (B) objective measure of wellbeing using the EORTC EQ-5D-5L instrument; (C) cancer diagnosis and treatment details; (D and E) statements about clinical research and CCTs, rated on Likert scales a; (F) questions relating to CCT participation and decision making; (G) questions specifically targeted at those patients who had previously participated in a CCT.

### Statistical Analysis

Descriptive statistics are presented as means, medians, interquartile ranges and percentages. Categorical data associations were assessed using Pearson’s X^2^ or Fisher’s exact (when numbers in cells were <5) statistical tests. Variables were tested against patient demographic characteristics (Table 1) and offer of participation to a clinical trial, to explore for differences that may influence decisions regarding participation in CCTs. No adjustments to significance levels were made. Only significant results at p-value ≤ 5% are presented. Other tests of associations were conducted but not presented in the results, as numbers were too small or they did not achieve statistical significance at p-value ≤ 5%. We performed a binomial regression to look at the effects of confounders. Due to small numbers, dimensions of wellbeing were collapsed into patients that had no problems and those that had any problem (i.e., slight + moderate + severe + unable were combined). EORTC EQ-5D-5L results are expressed as medians. Missing data are included in relevant tables but were excluded from the analysis. Denominators varied as some questions were only applicable for certain group(s) of patients. Data were analysed using STATA version 15.0 (StataCorp, College Station Texas).

## 3. Results

### 3.1. Demographic Details 

A total of 1089 adult questionnaires were returned. Patients in the sample had a median age of 60 years (IQR; 50–69 years). Most patients were female (64%) and married or in a long-term relationship (71%), with 16% of patients living alone. Most patients were Irish (94%) and native English speakers (96%). Over half (58%) of patients had below tertiary levels of education, 30% were in some form of employment and 60% lived in an urban setting (Table 1).

### 3.2. Measure of Wellbeing Using the EORTC EQ-5D-5L Instrument 

The median score on the visual analogue scale with endpoints of 100 being ‘best health you can imagine’ to zero being the ‘worst health you can imagine’ was 75 (IQR 60–85). We used the EQ-5D five dimensions (mobility, i.e., ability to walk, self-care, usual activity, pain/discomfort and anxiety/depression), scoring them into five levels (no problem, slight problem, moderate problem, severe problem and extreme problem). Dimensions were collapsed for analysis into two groups—patients with and without a problem. Most patients (70%) reported having no mobility problems (based on ability to walk) and levels of mobility problems increased with age (X^2^ = 22.69; *p* < 0.0001) and were slightly higher in widowed patients (X^2^ = 7.82; *p* = 0.050) even after adjusting for age. Self-care issues were reported in 10% of patients, with proportions slightly higher in those only educated to primary school level (X^2^ = 17.47; *p* = 0.015). Over half (55%) of patients reported having pain/discomfort and 38% conveyed anxiety/depression. Patients aged 41–60 years reported more pain/discomfort and younger patients (<50 years) had slightly more anxiety/depression (X^2^ = 14.51; *p* = 0.006, X^2^ = 39.00; *p* < 0.0001). Females had more anxiety and depression than males (X^2^ = 13.49; *p* < 0.0001). No statistical difference was observed between groups in carrying out the dimension ‘usual activity’ (Table 2).

### 3.3. Participation 

Of the 1089 patients, 66% reported never being offered the option to take part in a CCT and only 5% (34/741) of those not offered asked about taking part. Of the 345 patients that were offered/asked to take part in CCTs, 79% participated. The majority (87%) of patients that received an explanation about CCTs understood it and 70% found the patient information leaflet easy to understand. However, 16% of patients invited to participate in CCTs reported not receiving an information leaflet. Just under half (48%) of patients made their decision on whether to participate in a CCT by themselves, 35% used help/support and 5% indicated that they would have liked help but did not have it. The main source of help came from family members (84%). For 53% of patients, participation in CCTs impacted positively on their quality of life, while 3% reported a negative effect. Only 33 patients (12%) were given the results of the published CCT. Information booklets and having a patient navigator were the main preferences for decision aids that participants felt may have helped when making a decision about CCT participation (Table 3).

### 3.4. Patient Understanding of Concepts Used in Clinical Trials 

All patients were asked whether they understood the term “clinical or medical trial”—this follows finding from the IPPOSI report of the Irish public where misunderstanding between medical and clinical research was prevalent [15]. Based on a binary response (yes/no), 77% of all surveyed patients understood the term, and this compares with 73% of the Irish public. In the public study, awareness was lower in males, in younger age groups and in lower socioeconomic status groups. In our patient sample, we found no significant differences in understanding between males and females or between socioeconomic groups, but did find that levels of understanding of the term were lower in older (X^2^ = 10.72; *p* = 0.003), widowed (X^2^ = 12.68; *p* = 0.005) and patients living in small towns (X^2^ = 7.98; *p* = 0.018).

Understanding of CCT methodology and clinical equipoise is known to cause problems for the public. When asked whether a randomised trial treatment is decided by chance, 39% of patients thought it was not and an additional 24% did not know. There were differences in understanding by age (X^2^ = 30.23; *p* < 0.0001), ethnicity (X^2^ =6.70; *p* = 0.035) and marital status (X^2^ = 16.20; *p* = 0.013). Less than half (48%) understood that CCTs were not only an option when standard of care treatment had failed. Proportions that understood this was lower in older age groups (X^2^ = 44.85; *p* < 0.0001), in single and widowed patients (X^2^ = 19.37; *p* = 0.004) and the self-employed (X^2^ = 27.24; *p* = 0.007). Fifty-eight percent of all patients and 61% of previous CCT participants thought that their doctor would ensure better treatment in a CCT. This was slightly higher in those with only tertiary education and in older age groups (X^2^ = 22.08; *p* = 0.037, X^2^ = 45.38; *p* < 0.0001), (Table 4).

### 3.5. Factors Influencing Patient’s Decision to Participate in a Cancer Clinical Trial 

The opportunity to advance cancer research (78%), possibility of feeling better and living longer (78%) and an oncologist recommendation (73%) were all positive influencing factors reported by patients considering CCT participation. Fear of the unknown and perceived risk of side effects can be a barrier to CCT participation. We found that 35% of patients reported fear of more side effects, 31% feared harm or death and 22% had a fear of being a human guinea pig as concerns for CCT participation. Of those that reported these fear factors, proportions were slightly higher in females (X^2^ = 5.24; *p* = 0.022, X^2^ = 8.40; *p* = 0.004). Females also expressed more concerns about additional hospital visits and distance to travel to participate in CCTs. More younger (<50 years) than older patients feared harm or death and had more concerns about whether the CCT treatment would work (X^2^ =18.93; *p* = 0.001, X^2^ = 45.00; *p* < 0.0001), (Table 5).

### 3.6. Attitudes towards Cancer Clinical Trials 

After receiving factual information about clinical research, the Irish public showed a willingness to engage in CCTs, but only a minority were willing to engage in therapeutic trials [15]. In our study, 66% of patient participants thought that cancer clinical research was a good idea, but this was lower in those aged >65 years (X^2^ = 31.46; *p* < 0.0001), with this age group also less willing to participate in drug trials compared with younger patients. We found that 63% of patients would participate in CCTs with a new drug, but this was slightly lower (59%) for participation in a CCT with a new drug only tested on animals, and 70% indicated that they would be willing to participate in translational trials. More males than females would consider participating in CCTs to determine whether a new drug is better or the same as the usual treatment (X^2^ = 14.57; *p* = 0.001), on drug trials with no standard treatment-only animal data (X^2^ = 11.76; *p* = 0.003) and in translational trials (X^2^ = 7.48; *p* = 0.024). The majority (89%) of patients were willing to supply personal information, an issue of concern raised by the Irish public [15], Table 6.

### 3.7. Trust in Supports (All Patients)

Using a scale from 0 to 10, with a score of 0 indicating no trust and a score of 10 indicating maximum trust, patients rated information sources. Oncologists and research nurse specialists scored highest, with mean scores of 9.4 and 9.0, respectively. GPs and patient information booklets scored 7.8 and 7.5, respectively. The internet and newspaper/magazine reports were the least trusted, with scores of 3.7 and 3.8, respectively.

## 4. Discussion

Based on availability and patient eligibility, CCTs are a treatment option for patients. Before a patient chooses to participate in a clinical trial, they must first know that this is an option and understand clinical research. The aim of awareness ensures that patients can ask their oncology teams about CCT options, and doing so will ‘normalize’ CCTs as part of treatment option discussions. Increasing understanding and awareness may therefore also increase accrual. This national study aimed to describe understanding and awareness in Irish oncology patients after a previous study of the Irish public identified concerns around knowledge and understanding of cancer clinical research. Dissatisfaction by the public with the Irish health service and support by the Irish public for Ireland to operate as a focal point for clinical research provides an opportunity for clinical trials if the barriers to participation can be addressed [15].

We found slightly more oncology patients understood the term ‘clinical trial’ than the Irish public and while understanding of clinical research was lower in younger age groups in the general population [15], we found the opposite in cancer patients with older patients having poorer levels of understanding. There was great variation in understanding of cancer clinical trial methodology. Demographic and socioeconomic disparities are known to occur in clinical trial participation, with much lower recruitment of elderly patients [9,17,18]. We found uncertainty and a lack of understanding was more prevalent in older age groups, those that were widowed and in those with fewer years of education. Concern regarding treatment side effects is also a frequently reported reason for non-participation in CCTs [15,19,20,21]. Contrary to other studies and findings from the Irish public study [19,21,22], we found that over two-thirds of oncology patients in the survey did not report fear of more side effects, harm or death as being negative factors in their decision making around CCT participation. Where fears were present, this was higher in younger rather than older age groups and in females.

Two-thirds of oncology patients in our study were not offered the option to take part in a CCT and only 5% of patients not offered a trial independently asked about CCT participation, a finding consistent with other studies [22,23]. This outlines the need for better CCT awareness by patients and more incorporation of CCT discussion by medical teams with patients when treatment options are being discussed. Of those that took up the offer of a CCT, 79% participated and an additional 6% accepted but were not eligible, while only 3% declined the offer. Willingness to participate was positively influenced by prior participation. Logistical concerns are often cited as a barrier to participation [9], and 20–25% of patients in our study had some logistical concerns this was more prevalent in females than males.

Patients are known to struggle with the concepts of chance and randomisation, have uncertainty about the appropriateness of CCTs for serious illnesses, and consider CCTs as valid treatment options only when standard treatment has failed [20,24]. Our study concurred with these findings. We found that the number of patients having difficulty understanding these concepts were more prevalent in older age groups. Difficulty with the concept of randomisation is a recognised factor affecting accrual to CCTs [15,24,25,26,27]. Understanding and acceptability of clinical equipoise is important in determining whether patients consent to randomisation and accept the treatment allocation assigned to them [28]. In our study, 58% of patients and 60% of previous CCT participants had a “therapeutic misconception” [29], despite 87% of previous CCT participants reportedly understanding the trial explanation. This discrepancy underlines the importance of checking and ensuring patients knowledge and understanding of what they are being told. Consultations between patients and oncologists have highlighted failures to provide consistent sequences of information with rationale for randomising [30], and ensuring consistency in explanations of clinical trial methodology would address some of these discrepancies.

The pharmacy industry is the largest sponsor of clinical trials, mainly undertaken on unapproved drugs to support an application for marketing approval [9]. A principal barrier to CCT uptake expressed by the Irish public was participation in drug trials [15]. We found that almost two-thirds of oncology patients initially expressed a willingness to participate in drug clinical trials but previous CCT participants changed their decisions about taking part when reminded that the trial drug might be worse than the standard-of-care treatment. Females in the study showed slight indications of being more risk adverse and were less willing than males to participate in theoretical CCTs for new drugs and translational trials.

Most patients who had been offered CCT participation selected having an information booklet or patient navigator as aids that they would like to have to assist them in making their decision and 59% of patients preference was to have CCTs explained to them by their consultant. The patient’s family can have a significant influence on their decision-making process, with 84% of patients that reported using support relying on their family to assist them in making decisions about CCT participation. However, some studies have shown that targeted awareness campaigns focusing on newly diagnosed patients and their families have not shown any significant increases in trial enrolment [9].

The hope of a personally improved outcome and altruistic reasons such as helping to fight cancer are commonly cited reasons for CCT participation [22,23,24]. The most frequent reasons given in our study for positively considering CCT participation was to feel better and live longer. We also found that Irish oncology patients, like patients elsewhere, wanted to participate in clinical trials to advance cancer research [9].

Only 14% of patients indicated that they would feel too weak to participate in CCTs. Our study population had a slightly higher median EQ VAS score of 75 compared to other studies of cancer patients [31,32,33]. We also found that of the 38% of participants reported as having some level of anxiety/depression, this was more prevalent in younger age groups and in female patients.

Many of the barriers to CCT participation that we found in our study of Irish oncology patients are already known. We did, however, find lower levels of commonly cited reasons to decline involvement in CCTs such as fear of side effects. The lower levels of fear were contrary to what was expressed in the general population and may be indicative of patients having a better awareness of cancer treatment side effects. We also found that only a small proportion of patients asked about clinical trials, indicative of a lack of awareness and highlighting a much broader issue around patient–clinician communication. Many patients are at a disadvantage compared to clinical staff in not knowing about clinical trials availability and understanding the associated concepts and methodology and depend on their advice. Our principal finding was that elderly patients had consistently, across the variables tested, significantly less understanding of CCTs and would benefit from support. A lack of understanding of therapeutic trial methodology in the Irish population is likely to be a real barrier to uptake for these clinical trials and would no doubt benefit from a national communication campaign to raise awareness in this area.

There are several limitations to our study. The national accrual rate to CCTs is between 3 and 5%. Almost one-third of our study cohort reported being on a CCT, indicating selection bias in our sample. It is likely that CCT participants’ answers reflect real-life decisions that they made about participation. Patients often express a theoretical willingness/agreement to participate in hypothetical CCT scenarios and the study could not capture their actual decision in a real situation, and it is also possible that some patients gave socially desirable responses [24]. It is possible that despite provision of information, some patients may have confused being involved in a translational study with being on a CCT. We accept that missing data will also represent a source of bias. However, it is unlikely to affect the results due to random distribution across the variables. We excluded missing values when testing for associations.

## 5. Conclusions

Irish oncology patients understand the importance of cancer clinical research and having CCT options available to them. However, considerable misunderstanding regarding clinical equipoise and uncertainty around decision making was an issue, particularly in older patients. The global underrepresentation of older patients in clinical trials has not only been a health care disparity but has likely left this population with reduced understanding and awareness of clinical trials. Cancer incidence and mortality are growing worldwide, reflecting both an aging and rapidly growing global population. Advancing age is a known risk factor for cancer and it is imperative that older patients are represented in cancer clinical research and that awareness of clinical trials is raised in this population globally. Historically, clinical trials have been biased towards younger age groups.

Unlike in other studies, we found Irish cancerpatients had less concerns around known fears of barriersto CCT participation such as fear of more side-effects, harm or death. They also expressed high levels of trust in oncology clinicians and nurses yet had reservations about participating in therapeutic trials. A qualitative study to better understand the reasons for this would be beneficial. Patient willingness to participate in therapeutic trials could be positively influenced by more awareness around CCT methodology. We found that younger females were slightly more risk adverse when considering new drug trial scenarios, possibly influenced by their higher levels of anxiety. Recognising and addressing these concerns may reduce anxiety and improve willingness to participate in CCTs. Improvement in information delivery on CCT concepts is warranted and tailoring communication of CCT information would be beneficial, particularly for the groups highlighted in the study that had lower levels of understanding. Patients in the study indicated that having a patient navigator would be a useful aid and consideration of the use of trained facilitators to support communication of the importance of clinical research may be advantageous.

This is the first nationwide study of oncology patients’ attitudes and understanding of cancer clinical trials in Ireland, and the findings can contribute towards considered interventions for improvement to help optimize accrual rates.

## Figures and Tables

**Table 1:** Demographic Details.

*n* = 1089	Number (*n*)	Percentage (%)
Age (Years)	*Missing = 18*	*2*
Mean age	59 (min 18, max 89)	
Median age	60 (IQR 50–69, *n* = 1071)	
Median age female	57 (IQR 48–66, *n* = 690)	
Median age male	65 (IQR 56–71, *n* = 381)	
≤40	99	9
41–50	179	16
51–60	275	25
61–70	308	28
>71	210	19
≤65	708	65
>65	363	33
Gender	*Missing = 7*	*1*
Male	386	35
Female	696	64
Ethnicity *	*Missing = 8*	*1*
Irish	1019	94
Irish Traveller	2	<1
Other European	47	4
Asian	9	1
African	1	<1
Other	3	<1
Marital Status	*Missing = 7*	*1*
Married/living with partner	778	71
Divorced	27	2
Single	129	12
Widowed	97	9
Separated	51	5
Number in household	*Missing = 24*	*2*
Respondent only	170	16
+1	382	35
+2	210	19
+3	157	14
+4	89	8
+5	39	4
+6	13	1
+7	2	<1
+8	1	<1
+9	1	<1
+13	1	<1
Care for children<18 or elderly?	*Missing = 27*	*2*
Yes	268	25
No	794	73
If yes, how many do you care for?	*Missing = 5 (263 0f 268)*	*2*
0	2	1
1	110	41
2	98	37
3	34	13
4	17	6
5	2	1
Location ***	*Missing = 9*	*1*
City/Big town	436	40
Countryside/Village	419	38
Small town	215	20
Other	10	1
Number of cars in household	*Missing = 10*	*1*
0	120	11
1	499	46
≥2	460	42
Current accommodation	*Missing = 14*	*1*
Owned	572	53
Mortgage	309	28
Rent local authority	88	8
Rent private	73	7
Other	33	3
Education **	*Missing = 42*	*4*
No formal education	16	1
Primary school	160	15
Junior/Intermediate	213	20
Leaving certificate	236	22
Post-diploma/cert	203	19
University degree	106	10
Higher degree	113	10
Occupation	*Missing = 34*	*3*
Full time	157	14
Part time	83	8
Homemaker	143	13
Self-employed	84	8
Retired	344	32
Student	6	1
Sick, can’t work	173	16
Unemployed	47	4
Other	18	2
Earning status	*Missing = 197*	*18*
Main earner in 2 income house	95	9
Sole earner	93	9
Not main earner in 2 income house	135	12
Not applicable	569	52
Language	*Missing = 12*	*1*
English native language	1041	96
English not native, no translator required	30	3
English not native sometimes/always need translator	6	1

* Irish Travellers are a traditionally itinerant wayfaring ethnic group. ** Tertiary level education = third-level education (in the Irish context, below tertiary level includes the following groups: no formal education, primary school, junior/intermediate certificate, and leaving certificate). *** Location groupings were approximated to location of cancer centres.

**Table 2:** Wellbeing.

N = 1089	No Problem	Has Problem	Total	*X* ^2^	*p*
**Ability to walk (mobility)**	*n* (%)	*n* (%)	*n* (%)		
*Missing = 15*	766 (70)	308 (22)	1074 (98)		
**Age group**	*Missing = 32*				
<40 years	90 (92)	8 (8)	98 (100)	22.69	<0.0001
41–50 years	127 (72)	49 (28)	176 (100)		
51–60 years	191 (70)	82 (30)	273 (100)		
61–70 years	209 (69)	95 (31)	304 (100)		
>71 years	140 (68)	66 (32)	206 (100)		
Total	757 (72)	300 (28)	1057 (100)		
**Marital status**	*Missing = 20*				
Married	555 (72)	213 (28)	768 (100)	7.82	0.050
Separated/divorced	54 (69)	24 (31)	78 (100)		
Single	95 (75)	31 (35)	126 (100)		
Widowed	58 (60)	39 (40)	97 (100)		
Total	762 (71)	307 (29)	1069 (100)		
**Self-care**	964 (88)	110 (10)	1074 (98)	*Missing = 15*	
**Marital status**	*Missing = 20*				
Married	693 (90)	76 (10)	769 (100)	9.44	0.024
Separated/divorced	69 (90)	8 (10)	77 (100)		
Single	118 (94)	8 (6)	126 (100)		
Widowed	79 (81)	18 (19)	97 (100)		
Total	959 (90)	110 (10)	1069 (100)		
**Education**	*Missing = 57*				
No formal education	15 (100)	0 (0)	15 (100)	17.47	0.015
Primary school	129 (82)	29 (18)	158 (100)		
Junior/Intermediate cert	194 (92)	16 (8)	210 (100)		
Leaving cert	213 (92)	18 (8)	231 (100)		
Post-leaving dip/cert	180 (90)	20 (10)	200 (100)		
University—primary degree	97 (92)	9 (8)	106 (100)		
University—higher degree	97 (87)	15 (13)	112 (100)		
Total	925 (90)	107 (10)	1032 (100)		
**Pain/discomfort**	472 (43)	600 (55)	1072 (98)	*Missing = 17*	
**Age group**	*Missing = 33*				
<40 years	47 (47)	52 (53)	99 (100)	14.51	0.006
41–50 years	66 (37)	112 (63)	178 (100)		
51–60 years	102 (37)	170 (63)	272 (100)		
61–70 years	153 (51)	149 (49)	302 (100)		
>71 years	95 (46)	110 (54)	205 (100)		
Total	463 (44)	593 (56)	1056 (100)		
**Anxiety/depression**	650 (60)	420 (38)	1071 (98)	*Missing = 17*	
**Age group**	*Missing = 35*				
<40 years	49 (49)	50 (51)	99 (100)	39.00	<0.0001
41–50 years	90 (51)	87 (49)	177 (100)		
51–60 years	146 (54)	126 (46)	272 (100)		
61–70 years	205 (68)	98 (32)	303 (100)		
>71 years	150 (74)	53 (26)	203 (100)		
Total	640 (61)	414 (39)	1054 (100)		
**Sex**	*Missing = 25*				
Male	260 (68)	122 (32)	382 (100)	13.49	<0.0001
Female	386 (57)	296 (43)	682 (100)		
Total	646 (61)	418 (39)	1064 (100)		
**Employment**	*Missing = 77 (excl. student n = 6 and others n = 18)*				
Full time	100 (65)	55 (35)	155 (100)	12.88	0.045
Part time	46 (55)	37 (45)	83 (100)		
Homemaker	74 (52)	68 (48)	142 (100)		
Self-employed	53 (64)	30 (36)	83 (100)		
Retired	219 (66)	114 (34)	333 (100)		
Sick, can’t work	103 (60)	68 (40)	171 (100)		
Unemployed	22 (49)	23 (51)	45 (100)		
Total	617 (61)	395 (39)	1012 (100)		
**Visual scale of patient’s health 0–100, where 0 = worst health, 100 = best health**					
	**N**	**Mean**	**Median**	**IQR**	
Health	1038	72.6	75	60–85	

Mobility refers to ‘ability to walk’. Only results at p-value ≤5% are presented. Due to rounding, numbers and percentages may not add up precisely to totals. Analysis excludes missing numbers.

**Table 3:** Participation and Perception of Cancer Clinical Trials.

*Table Has Different Denominators, as Some Questions Were Only Relevant to Specific Groups, Depending on Previous Responses*		
	Number (*n*)	Percentage (%)
**F1. Were you offered the option to take part in a CCT? ?**	(*n* = 1089)	
**Yes**	311	29
**No**	716	66
**Do not remember**	25	2
**Missing**	*37*	*3*
**Total**	1089	100
***Question F2 based on ‘no’ and ‘don’t remember’ responses to F1***		
**F2. Didyou ever ask to take part in a CCT?**	(*n*= 741)	
**Yes**	34	5
**No**	700	94
**Missing**	7	1
**Total**	741	100
***Questions F3 to F8 based on those that responded yes in F1 + F2***		
**F3. The trial was explained to me by;^**	(*n* = 345)	
**My consultant**	206	60
**A junior doctor**	15	4
**A research nurse**	189	55
**Other**	18	5
**F4. Who should have explained CCTs to you? ^**	(*n* = 345)	
**My consultant**	204	59
**A junior doctor**	8	2
**Research nurse**	148	43
**Other**	9	3
**F5. Did you understand the explanation well?**	(*n* = 345)	
**Yes**	302	87
**No**	16	5
**Missing**	27	8
**Total**	345	100
**F6. Was the CCT information leaflet easy to understand?**	(*n* = 345)	
**Yes**	243	70
**No**	18	5
**Not offered leaflet**	55	16
**Missing**	29	9
**Total**	345	100
**F7. What decision aids would have helped? ^**	(*n* = 345)	
**Information booklet**	181	52
**Educational video**	59	17
**Patient navigator**	138	40
**Question prompt list**	81	23
**No decision aid required**	64	18
**F8. I made the decision about participation in the CCT by:**	(*n* = 345)	
**Myself without help**	167	48
**After using help or support**	121	35
**No help but would have liked help**	16	5
**Missing**	*41*	12
**Total**	345	100
***Questions F9 and F10 are based on respondents of F8 that reported using help/support***		
**F9. If you had help in F8, what sources did you use? ^**	(*n* = 121)	
**Family**	102	84
**GP**	31	26
**The internet**	15	12
**Cancer Support Centre**	19	16
**The Daffodil Centre**	3	2
**The Irish Cancer Society**	6	5
**Asking patients with the disease through an online support group**	5	4
**Other**	37	30
**F10. Did your support in F9**	(*n* = 121)	
**Encourage you to participate**	85	70
**Discourage you from participating**	1	1
**Neither encourage/discourage**	28	23
**Some encouraged/discouraged**	7	6
**Total**	121	100
***Questions F12 to F13 are based on those that responded yes in F1 + F2***		
**F12. What was the outcome of the offer to participate in the CCT?**	(*n* = 345)	
**Accepted offer and participated**	271	79
**Declined offer to participate**	12	3
**Accepted but was ineligible**	19	6
**Missing**	43	12
**Total**	345	100
**F13. Regarding your decision to take part in a CCT, were you satisfied with the decision?**	(*n* = 345)	
**Dissatisfied**	3	1
**Neither dissatisfied/satisfied**	29	8
**Satisfied**	267	77
**Missing**	46	13
**Total**	345	100
***Questions G1 to G8 are based on those that took part in a CCT (F12), n = 262***		
**G1. My experience on a CCT was positive**	(*n* = 271)	
**Strongly disagree**	0	0
**Disagree**	0	0
**Unsure**	15	6
**Agree**	93	34
**Strongly Agree**	131	48
**Missing**	32	12
**Total**	271	100
**G2. Myexperience on the CCT was what Iexpected**	(*n* = 271)	
**Yes**	216	80
**No**	22	8
**Missing**	*33*	12
**Total**	*271*	100
**G3. I would participate in a CCT again?**	(*n* = 271)	
**Yes**	233	86
**No**	4	1
**Missing**	34	13
**Total**	*271*	100
**G4. I would you recommend CCT participation to others**	(*n* = 271)	
**Yes**	231	85
**No**	6	2
**Missing**	34	13
**Total**	271	100
**G5. What was the effect of the CCT on your quality of life?**	(*n* = 271)	
**Positive affect**	145	53
**Negative affect**	8	3
**No affect**	89	33
**Missing**	29	11
**Total**	271	100
**G6. Was too much paperwork required for the CCT?**	(*n* = 271)	
**Yes**	24	9
**No**	216	80
**Missing**	31	11
**Total**	271	100
**G7. There were too many hospital visits involved in the CCT**	(*n* = 271)	
**Yes**	26	10
**No**	215	79
**Missing**	30	11
**Total**	271	100
**G8. I received the published results of the CCT?**	(*n* = 271)	
**Yes**	33	12
**No, but would like to have them**	174	64
**No and do not want to know**	25	9
**Missing**	*39*	15
**Total**	271	100

^ Participants may have selected more than one source, and therefore data will not add up to 100%. Table relates to sections ‘F and G’ in questionnaire Due to rounding numbers and percentages may not add up precisely to totals.

**Table 4:** Understanding of Clinical Trial Methodology.

*n* = 1089	Yes *n* (%)	No *n* (%)	Don’t Know *n* (%)	Total *n* (%)	*X* ^2^	*p*
**Do you understand the term “clinical or medical trial”?** ***Missing = 68***	840 (77)	181 (17)	-	1021 (94)		
**Age group**	*Missing = 83*					
<40 years	87 (90)	10 (10)	-	97 (100)	10.72	0.003
41–50 years	146 (84)	27 (16)	-	173 (100)		
51–60 years	221 (86)	37 (14)	-	258 (100)		
61–70 years	228 (80)	57 (20)	-	285 (100)		
>71 years	149 (77)	44 (23)	-	193 (100)		
Total	831 (83)	175 (17)	-	1006 (100)		
**Marital status**	*Missing = 72*					
Married	616 (85)	112 (15)	-	728 (100)	12.68	0.005
Separate/divorced	59 (81)	14 (19)	-	73 (100)		
Single	93 (77)	28 (23)	-	121 (100)		
Widowed	68 (72)	27 (28)	-	95 (100)		
Total	836 (82)	181 (18)	-	1017 (100)		
Location *	*Missing = 75, excludes ‘other’ category (n = 10)*					
City/Big town	355 (86)	60 (14)	-	415 (100)	7.98	0.018
Countryside/Village	322 (82)	70 (18)	-	392 (100)		
Small town	151 (76)	47 (24)	-	198 (100)		
Total	828 (82)	177 (18)	-	1005 (100)		
**In a RCT treatment is decided by chance** ***Missing = 64***	344 (32)	425 (39)	256 (24)	1025 (94)		
**Age group**	*Missing = 79*					
<40 years	45 (46)	44 (45)	9 (9)	98 (100)	30.23	<0.0001
41–50 years	67 (40)	67 (40)	35 (20)	169 (100)		
51–60 years	85 (32)	118 (44)	63 (24)	266 (100)		
61–70 years	88 (31)	123 (43)	75 (26)	286 (100)		
>71 years	56 (29)	68 (36)	67 (35)	191 (100)		
Total	341 (34)	420 (41)	249 (25)	1010 (100)		
**Ethnicity**	*Missing = 72*					
Irish	311 (32)	402 (42)	247 (26)	960 (100)	6.70	0.035
Others	27 (47)	22 (39)	8 (14)	57 (100)		
Total	338 (33)	424 (42)	255 (25)	1017 (100)		
**Marital status**	*Missing = 69*					
Married	256 (35)	316 (43)	161 (22)	733 (100)	16.20	0.013
Separate/divorced	22 (30)	29 (39)	23 (31)	74 (100)		
Single	36 (30)	50 (41)	35 (29)	121 (100)		
Widowed	26 (28)	30 (33)	36 (39)	92 (100)		
Total	340 (33)	425 (42)	255 (25)	1020 (100)		
**CCT only used when standard treatments have not worked** ***Missing = 63***	226 (21)	527 (48)	273 (25)	1026 (94)		
**Age group**	*Missing = 77*					
<40 years	15 (15)	67 (68)	17 (17)	99 (100)	44.85	<0.0001
41–50 years	31 (18)	107 (62)	34 (20)	172 (100)		
51–60 years	68 (26)	143 (54)	52 (20)	263 (100)		
61–70 years	64 (22)	129 (45)	95 (33)	288 (100)		
>71 years	44 (23)	76 (40)	70 (37)	190 (100)		
Total	222 (22)	522 (52)	268 (26)	1012 (100)		
**Marital status**	*Missing = 67*					
Married	163 (22)	393 (54)	178 (24)	734 (100)	19.37	0.004
Separate/divorced	7 (9)	41 (53)	28 (37)	75 (100)		
Single	30 (25)	60 (49)	32 (26)	122 (100)		
Widowed	23 (25)	34 (37)	34 (37)	91 (100)		
Total	223 (22)	527 (51)	272 (27)	1022 (100)		
**Employment**	*Missing = 119, (excl. student n = 6 and others n = 18)*					
Full time	21 (14)	86 (57)	43 (29)	150 (100)	7.24	0.007
Part time	17 (22)	42 (55)	18 (23)	77 (100)		
Homemaker	21 (16)	69 (51)	44 (33)	134 (100)		
Self-employed	25 (31)	33 (41)	22 (28)	80 (100)		
Retired	74 (23)	182 (57)	64 (20)	320 (100)		
Sick, can’t work	35 (22)	73 (45)	54 (33)	162 (100)		
Unemployed	12 (25)	21 (45)	14 (30)	47 (5)		
Total	205 (21)	506 (52)	259 (27)	970 (100)		
**CCTs test treatments that nobody knows anything about** ***Missing = 77***	143 (13)	607 (56)	262 (24)	1012 (93)		
**Age group**	*Missing = 91*					
<40 years	17 (17)	74 (75)	8 (8)	99 (100)	49.77	<0.0001
41–50 years	21 (13)	115 (68)	32 (19)	168 (100)		
51–60 years	34 (13)	173 (67)	53 (20)	260 (100)		
61–70 years	36 (13)	146 (52)	100 (35)	282 (100)		
>71 years	33 (17)	93 (49)	63 (33)	189 (100)		
Total	141 (14)	601 (60)	256 (26)	998 (100)		
**Marital status**	*Missing = 82*					
Married	84 (12)	457 (63)	182 (25)	723 (100)	21.36	0.002
Separate/divorced	14 (19)	37 (51)	21 (29)	72 (100)		
Single	23 (19)	72 (59)	27 (22)	122 (100)		
Widowed	21 (23)	39 (43)	30 (33)	90 (100)		
Total	142 (14)	605 (60)	260 (26)	1007 (100)		
Location **	*Missing = 85*					
City/Big town	55 (13)	255 (62)	100 (24)	410 (100)	13.68	0.033
Countryside/Village	46 (12)	237 (61)	106 (27)	389 (100)		
Small town	1 (12)	2 (25)	5 (63)	8 (100)		
Other	39 (20)	111 (56)	47 (24)	197 (100)		
Total	141 (14)	605 (60)	258 (26)	1004 (100)		
Uptake of CCT *	*NOTE the denominator = 345; missing = 58*					
Accepted offer and participated	30 (12)	191 (74)	37 (14)	258 (100)	15.88	0.015
Declined offer to participate	0 (0)	5 (45)	6 (55)	11 (100)		
Accepted but was ineligible	1 (6)	16 (89)	1 (6)	18 (100)		
Total	31 (11)	212 (74)	44 (15)	287 (100)		
**CCTs are not appropriate for diseases like cancer** ***Missing = 62***	59 (5)	751 (69)	217 (20)	1027 (94)		
**Age group**	*Missing = 76*					
<40 years	2 (2)	86 (87)	11 (11)	99 (100)	31.57	<0.0001
41–50 years	8 (5)	138 (81)	25 (15)	171 (100)		
51–60 years	12 (5)	206 (77)	48 (18)	266 (100)		
61–70 years	21 (7)	189 (67)	74 (26)	284 (100)		
>71 years	14 (7)	124 (64)	55 (29)	193 (100)		
Total	57 (6)	743 (73)	213 (21)	1013 (100)		
**My doctor would know which treatment in a CCT was better** ***Missing = 62***	581 (53)	209 (19)	237 (22)	1027 (94)		
**Age group**	*Missing = 77*					
<40 years	37 (38)	33 (34)	28 (28)	98 (100)	37.58	<0.0001
41–50 years	80 (47)	44 (26)	45 (27)	169 (100)		
51–60 years	147 (56)	61 (23)	56 (21)	264 (100)		
61–70 years	185 (64)	46 (16)	59 (20)	290 (100)		
>71 years	123 (64)	24 (13)	44 (23)	191 (100)		
Total	572 (57)	208 (20)	232 (23)	1012 (100)		
**Marital status**	*Missing = 67*					
Married	412 (56)	149 (20)	173 (24)	734 (100)	14.11	0.028
Separate/divorced	34 (46)	24 (32)	16 (22)	74 (100)		
Single	68 (56)	27 (22)	27 (22)	122 (100)		
Widowed	62 (67)	9 (10)	21 (23)	92 (100)		
Total	576 (56)	209 (21)	237 (23)	1022 (100)		
**My doctor would ensure that I get better treatment in a CCT** ***Missing = 50***	632 (58)	188 (17)	219 (20)	1039 (95)		
**Age group**	*Missing = 65*					
<40 years	39 (39)	30 (30)	30 (30)	99 (100)	45.38	<0.0001
41–50 years	89 (52)	44 (25)	39 (23)	172 (100)		
51–60 years	165 (62)	55 (20)	47 (18)	267 (100)		
61–70 years	190 (65)	38 (13)	64 (22)	292 (100)		
>71 years	138 (71)	20 (10)	36 (19)	194 (100)		
Total	621 (100)	187 (100)	216 (100)	1024 (100)		
Education ***	*Missing = 90*					
No formal	9 (60)	1 (7)	5 (33)	15 (100)	22.08	0.037
Primary school	98 (65)	25 (16)	29 (19)	152 (100)		
Junior/Intermediate cert	143 (72)	23 (11)	34 (17)	200 (100)		
Leaving cert	127 (57)	50 (22)	47 (21)	224 (100)		
Post-leaving dip/cert	121 (61)	39 (20)	38 (19)	198 (100)		
Primary degree	58 (58)	17 (17)	25 (25)	100 (100)		
Higher degree	56 (51)	26 (24)	28 (25)	110 (100)		
Total	612 (61)	181 (18)	206 (21)	999 (100)		
**Employment**	*Missing = 107 (excl. student n = 6 and others n = 18)*					
Full time	73 (49)	42 (28)	35 (23)	150 (100)	22.40	0.033
Part time	48 (61)	14 (18)	16 (21)	78 (100)		
Homemaker	85 (62)	24 (18)	27 (20)	136 (100)		
Self-employed	55 (67)	13 (16)	14 (17)	82 (100)		
Retired	218 (67)	46 (14)	63 (19)	327 (100)		
Sick, can’t work	93 (57)	26 (16)	43 (27)	162 (100)		
Unemployed	30 (64)	7 (15)	10 (21)	47 (100)		
Total	602 (61)	172 (18)	208 (21)	982 (100)		
**Uptake of CCT**	*NOTE the denominator = 345; missing = 80*					
Accepted offer and participated	162 (61)	66 (25)	37 (14)	265 (100)		
Declined offer to participate	6 (55)	3 (27)	2 (18)	11 (100)	8.70	0.044
Accepted but was ineligible	5 (28)	7 (39)	6 (33)	18 (100)		
Total	173 (59)	76 (26)	45 (15)	294 (100)		

* The ‘other’ category excluded in ‘Location’ due to small numbers. ** A total of 345 participants answered the outcome of being offered a CCT. RCT = randomised control trial, CCT = cancer clinical trial. *** Tertiary level education = third-level education (in the Irish context, below tertiary level includes the following groups: no formal education, primary school, junior/intermediate certificate, and leaving certificate)

**Table 5:** Factors Influencing Decision to Participate in Cancer Clinical Trials.

*n* = 1089	Yes (%)	No (%)	Total (%)	*X* ^2^	*p*
**Chance to advance cancer research to help others**	845 (78)	244 (22)	1089 (100)		
**Age group**	*Missing = 18*				
<40 years	91 (92)	8 (8)	99 (100)	45.29	<0.0001
41–50 years	152 (85)	27 (15)	179 (100)		
51–60 years	227 (83)	48 (17)	275 (100)		
61–70 years	227 (74)	81 (26)	308 (100)		
>71 years	135 (64)	75 (36)	210 (100)		
Total	832 (78)	239 (22)	1071 (100)		
**Marital status**	*Missing = 7*				
Married	622 (80)	156 (20)	778 (100)	29.66	<0.0001
Separate/divorced	62 (79)	16 (21)	78 (100)		
Single	102 (79)	27 (21)	129 (100)		
Widowed	54 (56)	43 (44)	97 (100)		
Total	840 (78)	242 (22)	1082 (100)		
**Education**	*Missing = 42*				
No formal	11 (69)	5 (31)	16 (100)	15.13	0.019
Primary school	117 (73)	43 (27)	160 (100)		
Junior/Intermediate cert	153 (72)	60 (28)	213 (100)		
Leaving cert	188 (80)	48 (20)	236 (100)		
Post-leaving dip/cert	174 (86)	29 (14)	203 (100)		
Primary degree	82 (77)	24 (23)	106 (100)		
Higher degree	85 (75)	28 (25)	113 (100)		
Total	810 (77)	237 (23)	1047 (100)		
**Uptake of CCT**	*NOTE the denominator = 345; missing = 43*				
Accepted offer and participated	235 (87)	36 (13)	271 (100)	7.12	0.028
Declined offer to participate	8 (67)	4 (33)	12 (100)		
Accepted but was ineligible	19 (100)	0 (0)	19 (100)		
Total	262 (100)	40 (100)	302 (100)		
**Chance might feel better/live longer**	850 (78)	239 (22)	1089 (100)		
**Age group**	*Missing = 18*				
<40 years	88 (89)	11 (11)	99 (100)	81.16	<0.0001
41–50 years	159 (89)	20 (11)	179 (100)		
51–60 years	231 (84)	44 (16)	275 (100)		
61–70 years	241 (78)	67 (22)	308 (100)		
>71 years	119 (57)	91 (43)	210 (100)		
Total	838 (78)	233 (22)	1071 (100)		
**Marital status**	*Missing = 7*				
Married	627 (81)	151 (19)	778 (100)	23.56	<0.0001
Separate/divorced	64 (82)	14 (18)	78 (100)		
Single	96 (74)	33 (26)	129 (100)		
Widowed	58 (60)	39 (40)	97 (100)		
Total	845 (78)	237 (22)	1082 (100)		
**Education**	*Missing = 42*				
No formal	12 (75)	4 (25)	16 (100)	12.63	0.049
Primary school	117 (73)	43 (27)	160 (100)		
Junior/Intermediate cert	154 (72)	59 (28)	213 (100)		
Leaving cert	184 (78)	52 (22)	236 (100)		
Post-leaving dip/cert	173 (85)	30 (25)	203 (100)		
Primary degree	84 (79)	22 (21)	106 (100)		
Higher degree	90 (80)	23 (20)	113 (100)		
Total	814 (78)	233 (22)	1047 (100)		
**Recommendation from cancer doctor**	797(73)	292 (27)	1089(100)		
**Age group**	*Missing = 18*				
<40 years	74 (75)	25 (25)	99 (100)	12.00	0.017
41–50 years	138 (77)	41 (23)	179 (100)		
51–60 years	211 (77)	64 (23)	275 (100)		
61–70 years	229 (74)	79 (26)	308 (100)		
>71 years	135 (64)	75 (36)	210 (100)		
Total	787 (73)	284 (27)	1071 (100)		
**Location**	*Missing = 19, excludes ‘other’ n = 10*				
City/Big town	302 (69)	134 (31)	436 (100)	7.87	0.020
Countryside/Village	314 (75)	105 (25)	419 (100)		
Small town	170 (79)	45 (21)	215 (100)		
Total	786 (73)	284 (27)	1070 (100)		
**Recommendation from GP**	360 (33)	729 (67)	1089 (100)		
**Gender**	*Missing = 7*				
Male	143 (37)	243 (63)	386 (100)	4.25	0.039
Female	215 (31)	481 (69)	696 (100)		
Total	358 (33)	724 (67)	1082 (100)		
**Uptake of CCT**	*NOTE the denominator = 345; missing = 43*				
Accepted offer and participated	96 (35)	175 (65)	271 (100)	7.66	0.012
Declined offer to participate	3 (25)	9 (75)	12 (100)		
Accepted but was ineligible	1 (5)	18 (95)	19 (100)		
Total	100 (33)	202 (67)	302 (100)		
**Previous experience on clinical trial**	199 (18)	890 (82)	1089 (100)		
**Age group**	*Missing = 18*				
<40 years	13 (13)	86 (87)	99 (100)	10.14	0.038
41–50 years	43 (24)	136 (76)	179 (100)		
51–60 years	57 (21)	218 (79)	275 (100)		
61–70 years	57 (19)	251 (81)	308 (100)		
>71 years	28 (13)	182 (87)	210 (100)		
Total	198 (18)	873 (82)	1071 (100)		
**Uptake of CCT**	*NOTE the denominator = 345; missing = 43*				
Accepted offer and participated	113 (42)	158 (58)	271 (100)	8.29	0.012
Declined offer to participate	3 (25)	9 (75)	12 (100)		
Accepted but was ineligible	2 (11)	17 (89)	19 (100)		
Total	118 (39)	184 (61)	302 (100)		
**Closer monitoring on clinical trial**	527 (48)	562 (52)	1089 (100)		
**Age group**	*Missing = 18*				
<40 years	49 (49)	50 (51)	99 (100)	37.83	<0.0001
41–50 years	106 (59)	79 (41)	179 (100)		
51–60 years	152 (55)	123 (45)	275 (100)		
61–70 years	148 (48)	160 (52)	308 (100)		
>71 years	66 (31)	144 (69)	210 (100)		
Total	521 (49)	550 (51)	1071 (100)		
**Marital status**	*Missing = 7*				
Married	400 (51)	378 (49)	778 (100)	22.88	<0.0001
Separate/divorced	36 (46)	42 (54)	78 (100)		
Single	63 (49)	66 (51)	129 (100)		
Widowed	25 (26)	72 (74)	97 (100)		
Total	524 (48)	558 (52)	1082 (100)		
**Education**	*Missing = 52*				
No formal	7 (44)	9 (56)	16 (100)	14.30	0.026
Primary school	74 (47)	82 (53)	156 (100)		
Junior/Intermediate cert	93 (44)	118 (56)	211 (100)		
Leaving cert	96 (41)	138 (59)	234 (100)		
Post-leaving dip/cert	117 (58)	85 (42)	202 (100)		
Primary degree	53 (50)	53 (50)	106 (100)		
Higher degree	56 (50)	56 (50)	112 (100)		
Total	496 (48)	541 (52)	1037 (100)		
**Fear more side effects**	300 (35)	709 (73)	1089 (100)		
**Age group**	*Missing = 18*				
<40 years	45 (45)	54 (55)	99 (100)	18.93	0.001
41–50 years	81 (45)	98 (55)	179 (100)		
51–60 years	92 (33)	183 (66)	275 (100)		
61–70 years	101 (33)	207 (67)	308 (100)		
>71 years	58 (28)	152 (72)	210 (100)		
Total	377 (35)	694 (65)	1071 (100)		
**Gender**	*Missing = 7*				
Male	118 (31)	268 (69)	386 (100)	5.24	0.022
Female	261 (36)	435 (63)	696 (100)		
Total	379 (35)	703 (65)	1082 (100)		
**Uptake of CCT**	*NOTE the denominator = 345; missing = 43*				
Accepted offer and participated	69 (25)	202 (75)	271 (100)	6.48	0.040
Declined offer to participate	7 (58)	5 (42)	12 (100)		
Accepted but was ineligible	6 (32)	13 (68)	19 (100)		
Total	82 (27)	220 (73)	302 (100)		
**Fear harm/death**	336 (31)	753 (69)	1089 (100)		
**Age group**	*Missing = 18*				
<40 years	46 (46)	53 (54)	99 (100)	24.26	<0.0001
41–50 years	69 (39)	110 (61)	179 (100)		
51–60 years	82 (30)	193 (70)	275 (100)		
61–70 years	85 (28)	223 (72)	308 (100)		
>71 years	48 (23)	162 (77)	210 (100)		
Total	330 (31)	741 (69)	1071 (100)		
**Location**	*Missing = 19, excludes ‘other’ n = 10*				
City/Big town	132 (30)	304 (70)	436 (100)	5.90	0.052
Countryside/Village	119 (28)	300 (72)	419 (100)		
Small town	81 (38)	134 (62)	215 (100)		
Total	332 (31)	738 (69)	1070 (100)		
**Education**	*Missing = 42*				
No formal	9 (56)	7 (44)	16 (100)	18.99	0.004
Primary school	63 (39)	97 (61)	160 (100)		
Junior/Intermediate cert	48 (23)	165 (77)	213 (100)		
Leaving cert	73 (31)	163 (69)	236 (100)		
Post-leaving dip/cert	56 (28)	147 (72)	203 (100)		
Primary degree	32 (30)	74 (70)	106 (100)		
Higher degree	39 (35)	74 (65)	113 (100)		
Total	320 (31)	727 (69)	1047 (100)		
**Uptake of CCT**	*NOTE the denominator = 345; missing = 43*				
Accepted offer and participated	66 (24)	205 (76)	271 (100)	6.42	0.040
Declined offer to participate	6 (50)	6 (50)	12 (100)		
Accepted but was ineligible	8 (42)	11 (58)	19 (100)		
Total	80 (26)	222 (74)	302 (100)		
**Fear of being a human guinea pig**	235 (22)	854 (78)	1089 (100)		
**Gender**	*Missing = 7*				
Male	65 (17)	321 (83)	386 (100)	8.40	0.004
Female	170 (24)	526 (76)	696 (100)		
Total	235 (22)	847 (78)	1082 (100)		
**Concerns about whether the new treatment works**	446 (41)	643 (59)	1089 (100)		
**Age group**	*Missing = 18*				
<40 years	63 (64)	36 (36)	99 (100)	45.00	<0.0001
41–50 years	87 (49)	92 (51)	179 (100)		
51–60 years	116 (42)	159 (58)	275 (100)		
61–70 years	115 (37)	193 (63)	308 (100)		
>71 years	56 (27)	154 (73)	210 (100)		
Total	437 (41)	634 (59)	1071 (100)		
**Marital status**	*Missing = 7*				
Married	311 (40)	467 (60)	778 (100)	9.66	0.022
Separate/divorced	31 (40)	47 (60)	78 (100)		
Single	68 (53)	61 (47)	129 (100)		
Widowed	33 (34)	64 (66)	97 (100)		
Total	443 (41)	639 (59)	1082 (100)		
**Education**	*Missing = 42*				
No formal	4 (25)	12 (75)	16 (100)	12.89	0.045
Primary school	77 (48)	83 (52)	160 (100)		
Junior/Intermediate cert	75 (35)	138 (65)	213 (100)		
Leaving cert	92 (39)	144 (61)	236 (100)		
Post-leaving dip/cert	96 (47)	107 (53)	203 (100)		
Primary degree	39 (37)	67 (63)	106 (100)		
Higher degree	43 (38)	70 (62)	113 (100)		
Total	426 (41)	621 (59)	1047 (100)		
**Feel too weak to take part**	156 (14)	933 (86)	1089 (100)		
**Gender**	*Missing = 7*				
Male	40 (10)	346 (90)	386 (100)	7.99	0.005
Female	116 (17)	580 (83)	696 (100)		
Total	156 (14)	926 (86)	1082 (100)		
**Time commitment**	163 (15)	926 (85)	1089 (100)		
**Ethnicity**	*Missing = 11*				
Irish	148 (15)	871 (85)	1019 (100)	3.70	0.054
Others	14 (24)	45 (76)	59 (100)		
Total	162 (15)	916 (85)	1078 (100)		
**Concern that there will be more hospital visits**	291 (27)	798 (73)	1089 (100)		
**Gender**	*Missing = 7*				
Male	80 (21)	306 (79)	386 (100)	11.30	0.001
Female	210 (30)	486 (70)	696 (100)		
Total	290 (27)	792 (73)	1082 (100)		
**Concern over the distance to travel**	194 (18)	895 (82)	1089 (100)		
**Gender**	*Missing = 7*				
Male	54 (14)	332 (86)	386 (100)	6.06	0.014
Female	139 (20)	557 (80)	696 (100)		
Total	193 (18)	889 (82)	1082 (100)		
**Location**	*Missing = 19, excludes ‘other’ n = 10*				
City/Big town	59 (14)	377 (86)	436 (100)	9.60	0.008
Countryside/Village	85 (20)	334 (80)	419 (100)		
Small town	47 (22)	168 (78)	215 (100)		
Total	191 (18)	879 (82)	1070 (100)		
**Needed more information**	411 (38)	678 (62)	1089 (100)		
**Age group**	*Missing = 18*				
<40 years	44 (44)	55 (56)	99 (100)	11.66	0.020
41–50 years	80 (45)	99 (55)	179 (100)		
51–60 years	110 (40)	165 (60)	275 (100)		
61–70 years	99 (32)	209 (68)	308 (100)		
>71 years	71 (34)	139 (66)	210 (100)		
Total	404 (38)	667 (62)	1071 (100)		
**Gender**	*Missing = 7*				
Male	121 (31)	265 (69)	386 (100)	10.34	0.001
Female	287 (41)	409 (59)	696 (100)		
Total	408 (38)	674 (62)	1082 (100)		

Yes, corresponds to number of patients that chose this factor, No corresponds to the remainder of patients in the sample.

**Table 6:** Willingness to Participate in Cancer Clinical Research.

*n* = 1089	Strongly Agree/Agree (%)	Strongly Disagree/Disagree (%)	Unsure (%)	Total (%)	*X* ^2^	*p*
**CCR to develop new ways to treat cancer is a good idea** *Missing = 32*	716 (66)	70 (6)	271 (25)	1057 (97)		
**Age group**	*Missing = 47*					
≤65 years	508 (72)	26 (4)	166 (24)	700 (100)	31.46	<0.0001
>65 years	203 (59)	40 (12)	99 (29)	342 (100)		
Total	711 (68)	66 (6)	265 (25)	1042 (100)		
**Uptake of CCT**	*NOTE the denominator = 345; missing = 59*					
Accepted offer and participated	245 (92)	3 (1)	17 (6)	265 (100)	30.63	<0.0001
Declined offer to participate	5 (45)	1 (9)	5 (45)	11 (100)		
Accepted but was ineligible	8 (80)	1 (10)	1 (10)	10 (100)		
Total	258 (90)	5 (2)	23 (8)	286 (100)		
**Marital status**	*Missing = 36*					
Married	519 (68)	46 (6)	194 (26)	759 (100)	20.50	0.011
Separate/divorced	59 (76)	3 (4)	16 (20)	78 (100)		
Single	85 (67)	5 (4)	37 (29)	127 (100)		
Widowed	51 (57)	15 (17)	23 (26)	89 (100)		
Total	714 (68)	69 (6)	270 (26)	1053 (100)		
**Willing to donate blood for CCR** *Missing = 33*	864 (79)	70 (6)	122 (11)	1056 (97)		
**Age group**	*Missing = 49*					
≤65 years	596(86)	27(4)	73(10)	696(100)	23.92	<0.0001
>65 years	261(76)	39(11)	44(13)	344(100)		
Total	857(82)	66(6)	117(11)	1040(100)		
**Gender**	*Missing = 39*					
Male	322 (86)	26 (7)	28 (7)	376 (100)	9.18	0.010
Female	539 (80)	43 (6)	92 (14)	674 (100)		
Total	861 (82)	69 (7)	120 (11)	1050 (100)		
**Uptake of CCT**	*NOTE the denominator = 345; missing = 50*					
Accepted offer and participated	246 (93)	5 (2)	13 (5)	264 (100)	13.24	0.017
Declined offer to participate	8 (67)	1 (8)	3 (25)	12 (100)		
Accepted but was ineligible	18 (95)	1 (5)	0 (0)	19 (100)		
Total	272 (92)	7 (2)	16 (5)	295 (100)		
**Willing to supply personal information for CCR** *Missing = 27*	969 (89)	36 (3)	57 (5)	1062 (97)		
**Location**	*Missing = 44*					
City/Big town	397 (93)	11 (3)	19 (4)	427 (100)	18.89	0.001
Countryside/Village	381 (93)	8 (2)	20 (5)	409 (100)		
Small town	176 (84)	15 (7)	18 (9)	209 (100)		
Total	954 (91)	34 (3)	57 (5)	1045 (100)		
**Would participate in a CCT with a new drug compared to standard** *Missing = 36*	687 (63)	67 (6)	299 (27)	1053 (97)		
**Age group**	*Missing = 52*					
≤65 years	477 (68)	26 (4)	193 (28)	696 (100)	21.92	<0.0001
>65 years	204 (60)	37 (11)	100 (29)	341 (100)		
Total	681 (66)	63 (6)	293 (28)	1037 (100)		
**Gender**	*Missing = 42*					
Male	271 (73)	21 (6)	81 (22)	373 (100)	14.57	0.001
Female	412 (61)	45 (7)	217 (32)	674 (100)		
Total	683 (65)	66 (6)	298 (28)	1047 (100)		
**Marital status**	*Missing = 41*					
Married	508 (68)	39 (5)	204 (27)	751 (100)	14.34	0.026
Separate/divorced	49 (64)	3 (4)	24 (32)	76 (100)		
Single	76 (60)	12 (9)	39 (31)	127 (100)		
Widowed	52 (55)	12 (13)	30 (32)	94 (100)		
Total	685 (65)	66 (6)	297 (28)	1048 (100)		
**Uptake of CCT**	*NOTE the denominator = 345; missing = 45*					
Accepted offer and participated	214 (80)	9 (3)	46 (17)	269 (100)	21.01	0.001
Declined offer to participate	3 (25)	1 (8)	8 (67)	12 (100)		
Accepted but was ineligible	16 (84)	0 (0)	3 (16)	19 (100)		
Total	233 (78)	10 (3)	57 (19)	300 (100)		
**Would participate in a CCT with a new drug only tested on animals** *Missing = 36*	641 (59)	76 (7)	336 (31)	1053 (97)		
**Age group**	*Missing = 53*					
≤65 years	454 (66)	26 (4)	213 (31)	693 (100)	42.83	<0.0001
>65 years	178 (52)	49 (14)	116 (34)	343 (100)		
Total	632 (61)	75 (7)	329 (32)	1036 (100)		
**Gender**	*Missing = 42*					
Male	251 (67)	19 (5)	102 (27)	372 (100)	11.76	0.003
Female	385 (57)	57 (8)	233 (35)	675 (100)		
Total	636 (61)	76 (7)	335 (32)	1047 (100)		
**Marital status**	*Missing = 41*					
Married	471 (62)	47 (6)	236 (31)	754 (100)	26.64	0.001
Separate/divorced	44 (59)	2 (3)	29 (39)	75 (100)		
Single	79 (63)	9 (7)	37 (30)	125 (100)		
Widowed	44 (47)	18 (19)	32 (34)	94 (100)		
Total	638 (61)	76 (7)	334 (32)	1048 (100)		
**Uptake of CCT**	*NOTE the denominator = 345; missing = 45*					
Accepted offer and participated	188 (70)	7 (3)	74 (27)	269 (100)	13.48	0.023
Declined offer to participate	5 (42)	2 (17)	5 (42)	12 (100)		
Accepted but was ineligible	17 (89)	0 (0)	2 (11)	19 (100)		
Total	210 (70)	9 (3)	81 (27)	300 (100)		
**Would participate in translational trials** *Missing = 37*	758 (70)	49 (5)	245 (22)	1052 (97)		
**Age group**	*Missing = 54*					
≤65 years	529 (76)	21 (3)	143 (21)	693 (100)	20.71	<0.0001
>65 years	221 (65)	27 (8)	94 (27)	342 (100)		
Total	750 (72)	48 (5)	237 (23)	1035 (100)		
**Gender**	*Missing = 43*					
Male	286 (77)	15 (4)	70 (19)	371 (100)	7.48	0.024
Female	467 (69)	34 (5)	174 (26)	675 (100)		
Total	753 (72)	49 (5)	244 (23)	1046 (100)		
**Marital status**	*Missing = 42*					
Married	557 (74)	33 (4)	163 (22)	753 (100)	19.67	0.003
Separate/divorced	56 (75)	1 (1)	18 (24)	75 (100)		
Single	87 (69)	4 (3)	35 (28)	126 (100)		
Widowed	54 (58)	11 (12)	28 (30)	93 (100)		
Total	754 (72)	49 (5)	244 (23)	1047 (100)		
**Uptake of CCT**	*NOTE the denominator = 345; missing = 43*					
Accepted offer and participated	231 (85)	1 (<1)	39 (14)	271 (100)	35.73	0.002
Declined offer to participate	6 (50)	2 (17)	4 (33)	12 (100)		
Accepted but was ineligible	15 (79)	0 (0)	4 (21)	19 (100)		
Total	252 (83)	3 (1)	47 (16)	302 (100)		

CCR = cancer clinical research; CCT = cancer clinical trial.
